# Effect of Selenium and Iodine on Oxidative Stress in the First Trimester Human Placenta Explants

**DOI:** 10.3390/nu13030800

**Published:** 2021-02-28

**Authors:** Nahal Habibi, Agatha Labrinidis, Shalem Yiner-Lee Leemaqz, Tanja Jankovic-Karasoulos, Dylan McCullough, Jessica A. Grieger, Sarah Gilbert, Carmela Ricciardelli, Shao Jia Zhou, Anthony V. Perkins, Claire T. Roberts, Tina Bianco-Miotto

**Affiliations:** 1 School of Agriculture, Food and Wine, Waite Research Institute, and Robinson Research Institute, University of Adelaide, Adelaide, SA 5005, Australia; nahal.habibi@adelaide.edu.au (N.H.); jo.zhou@adelaide.edu.au (S.J.Z.); 2 Adelaide Microscopy, Division of Research and Innovation, University of Adelaide, Adelaide, SA 5005, Australia; agatha.labrinidis@adelaide.edu.au (A.L.); sarah.gilbert@adelaide.edu.au (S.G.); 3 Flinders Health and Medical Research Institute, Flinders University, Bedford Park, SA 5042, Australia; shalem.leemaqz@flinders.edu.au (S.Y.-L.L.); tanja.jankovickarasoulos@flinders.edu.au (T.J.-K.); dylan.mccullough@flinders.edu.au (D.M.); claire.roberts@flinders.edu.au (C.T.R.); 4 Adelaide Medical School and Robinson Research Institute, University of Adelaide, Adelaide, SA 5005, Australia; jessica.grieger@adelaide.edu.au (J.A.G.); carmela.riccardelli@adelaide.edu.au (C.R.); 5 School of Medical Science, Griffith University, Gold Coast Campus, Parklands Drive, Southport, QLD 9726, Australia; a.perkins@griffith.edu.au

**Keywords:** apoptosis, copper, DNA damage, iodine, micronutrient supplement, oxidative stress, placenta, pregnancy, selenium

## Abstract

Imbalanced maternal micronutrient status, poor placentation, and oxidative stress are associated with greater risk of pregnancy complications, which impact mother and offspring health. As selenium, iodine, and copper are essential micronutrients with key roles in antioxidant systems, this study investigated their potential protective effects on placenta against oxidative stress. First trimester human placenta explants were treated with different concentrations of selenium (sodium selenite), iodine (potassium iodide), their combination or copper (copper (II) sulfate). The concentrations represented deficient, physiological, or super physiological levels. Oxidative stress was induced by menadione or antimycin. Placenta explants were collected, fixed, processed, and embedded for laser ablation inductively coupled plasma-mass spectrometry (LA ICP-MS) element imaging or immunohistochemical labelling. LA ICP-MS showed that placenta could uptake selenium and copper from the media. Sodium selenite and potassium iodide reduced DNA damage and apoptosis (*p* < 0.05). Following oxidative stress induction, a higher concentration of sodium selenite (1.6 µM) was needed to reduce DNA damage and apoptosis while both concentrations of potassium iodide (0.5 and 1 µM) were protective (*p* < 0.05). A high concentration of copper (40 µM) increased apoptosis and DNA damage but this effect was no longer significant after induction of oxidative stress. Micronutrients supplementation can increase their content within the placenta and an optimal maternal micronutrient level is essential for placenta health.

## 1. Introduction

Maternal nutrition during early pregnancy is critical for fetal growth and development and can impact the future health of the offspring later in life [1,2]. A deficiency or excess in micronutrients such as selenium, copper, and iodine measured in maternal plasma, serum, or urine are associated with a greater risk of pregnancy complications such as preeclampsia, gestational diabetes mellitus, spontaneous preterm birth, and small-for-gestational age [3,4,5,6,7]. The detrimental effects of pregnancy complications are not limited to the pregnancy as their long-term consequences can impact the health of both mother and child later in life [8,9,10]. Thus, it is of paramount importance to determine the nutritional risk factors related to pregnancy complications and devise potential preventive strategies.

The placenta is the key mediator of maternal nutrient supply to the fetus. Poor placental development, including incomplete remodelling of arteries during early pregnancy, has been associated with pregnancy complications like preeclampsia [11]. Partial remodelling of the maternal arteries causes hypoxia followed by reoxygenation that result in oxidative stress [12]. Oxidative stress is frequently associated with a range of pregnancy complications [13,14]. Oxidative stress is caused by accumulated reactive oxygen molecules and insufficient antioxidant activity [15,16], and results in damage of molecules such as lipids, proteins and DNA; thus, tissue decay [16]. In addition, oxidative stress can increase apoptosis and this leads to high shedding of syncytial fragments into maternal blood resulting in a systemic inflammatory response, which is associated with pregnancy complications [17]. 

Micronutrients and oxidative stress are related through the antioxidant system [18,19]. For instance, glutathione peroxidase (GPx) and superoxide dismutase 1 (SOD1) are two antioxidant enzymes that require micronutrients for proper function [18,19]. GPx needs selenium and SOD1 requires copper or zinc to scavenge radicals and prevent oxidative stress [18,19]. Studying micronutrient status in relation to placental development and oxidative stress may help elucidate the mechanisms involved in pregnancy complications. Although population studies have shown that selenium supplementation is associated with a lower incidence of preeclampsia and premature rupture of membranes [20,21,22], the impact of micronutrients on placental development is still unknown. In addition, there is no information on potential interactions between selenium and other micronutrients. Iodine is an essential micronutrient and its deficiency during pregnancy puts both mother and offspring at a greater risk of pregnancy complications such as gestational hypertension, intrauterine growth restriction and preterm birth [7,23,24,25,26,27]. We recently showed that selenium and iodine deficiency are associated with lower cell proliferation and higher cell death and lipid peroxidation in HTR8/SVneo trophoblast cells (Habibi et al., 2020). In addition, individual or combined supplementation with selenium and iodine protected trophoblast cells against oxidative stress by enhancing cell viability and proliferation and reducing lipid peroxidation [28]. 

Copper is another essential component in a variety of metalloenzymes [29] including the antioxidant SOD1 enzyme [18]. High copper levels in maternal serum have been associated with a greater level of inflammation [3]. Investigating how copper may impact the placenta will help to understand its role and impact on pregnancy outcome. This study investigated how selenium, iodine, and copper may affect oxidative stress response in first trimester human placenta by assessing their effect on proliferation, apoptosis, and DNA damage.

## 2. Materials and Methods

### 2.1. Placenta Explant Tissue Culture

First trimester (7–12 weeks’ gestation; determined from last menstrual period) human placenta tissue samples (*n* = 15) were collected with informed consent from women who underwent elective pregnancy terminations at the Pregnancy Advisory Centre, Woodville, South Australia. The age and BMI (mean ± SD) of participants were 28.83 ± 7.18 years and 25.36 ± 3.38 kg/m^2^, respectively. Samples were collected from participants that were Caucasian and terminations were not due to medical issues.

Within an hour of termination, 10–15 mg of placenta tissue sections (3–4 pieces) were cultured on a pre-prepared gel base in each well of a 48-well plate. The gel base was Growth Factor Reduced Matrigel^®^ (protein concentration: 3 mg/mL; Corning^®^) and 1X DMEM GlutaMAX™ media (Gibco^®^ Life Technologies™) containing 10% *v*/*v* FBS (Sigma-Aldrich^®^) and 1% *v*/*v* Antibiotic–Antimycotic (Life Technologies™) at pH 7.0. Culture conditions were maintained at 37 °C, 5% CO^2^, and 1% O^2^ for the duration of the experiments. Placenta explants were first cultured for 48 h to enable regeneration of the syncytial layer. 

Explants were supplemented with sodium selenite (Sigma Aldrich^®^) (0, 0.8, or 1.6 µM), potassium iodide (Sigma Aldrich^®^) (0, 0.5, or 1 µM), copper (II) sulfate (Sigma Aldrich^®^) (0, 20, or 40 µM), a combination of sodium selenite and potassium iodide (0.8 µM sodium selenite and 0.5 µM potassium iodide), or sterile Milli-Q water for 72 h with supplementation replenished every 24 h. These concentrations represent the low, physiological and supraphysiological level as measured in maternal blood during pregnancy [3,30]. 

Explants were then treated with 120 µM menadione, 480 µM antimycin or 0.1% ethanol (vehicle for menadione and antimycin) for 24 h. Explants were harvested and fixed in 10% neutral buffered formalin for 2 h at room temperature, then washed 3 times with 1X phosphate buffered saline (PBS) for 24 h at 4 °C and stored in 70% ethanol at 4 °C until processing. Explants were processed and embedded in paraffin blocks for downstream assessments. 

### 2.2. Micronutrient Uptake

To evaluate if placenta tissue explants take up selenium and copper, three first trimester placentas from 7 to 12 weeks’ gestation were cultured for 5 days including 48 h syncytial layer regeneration, and 72 h of supplementation with (0, 0.8, and 1.6 µM) sodium selenite or (0, 20, or 40 µM) copper (II) sulfate as above. Explants were then harvested, fixed, and paraffin embedded. Tissue sections of 10 µm were placed on microscope slides. Sections were heated for 2 h at 60 °C, then dewaxed with 100% xylene and 100% ethanol and washed with PBS two times and finally air dried overnight. Standards were made by dissolving 10% gelatine (Sigma Aldrich^®^) in elements solutions of selenium and copper with 0, 1, 10, and 100 mM concentrations. Gelatine blocks were mounted in Tissue-Tek OCT Compound medium. Gelatine blocks were cut in 10 µm sections to match placenta explant sections using a cryomicrotome (Leica CM3050s Cryostat). After placing gelatine sections on the microscope slides, they were kept at −20 °C. Gelatine sections were air dried overnight before laser ablation (LA) inductively coupled plasma-mass spectrometry (ICP-MS) analyses (Adelaide Microscopy, University of Adelaide), using an attached Resolution 193 nm excimer laser ablation system coupled to an Agilent 7900x ICP-MS. Samples were ablated with a series of parallel lines with the following conditions: 23 μm spots size, 23 μm/s speed, 10 Hz repetition rate, and a fluence of ~1 J/cm^2^. Intensity of micronutrients were recorded as counts per second for the following isotopes: 13C, 23Na, 24Mg, 29Mg, 31P, 39K, 43Ca, 57Fe, 65Cu, 66Zn, and 77Se. Only the elements of most interest (Se and Cu) are discussed in detail. Data was processed using the iolite data processing software, and concentrations were calculated and visualised relative to the gelatine standards to form a 2D map of micronutrient concentration over a chosen surface of each placenta explant sample.

### 2.3. Assessment of Proliferation, Apoptosis and DNA Damage 

To assess the effect of supplementation on placental proliferation, apoptosis and DNA damage, explants from first trimester placentas (*n* = 12) of 7–12 weeks’ gestation were cultured as mentioned above. Tissue sections of 5 µm were placed on microscope slides for immunohistochemical labelling for assessment of proliferation (Ki67; Abcam^®^; ab16667), apoptosis (cleaved caspase-3; Cell Signalling Technology^®^; CST.9661L) or DNA damage (8-hydroxy-2’-deoxyguanosine; Abcam^®^; ab48508) (Table 1). A Hamamatsu NanoZoomer Digital Pathology slide scanner was used to scan the stained sections. Eight areas per explant tissue, randomly chosen using NDP.view2 software, were used for quantification and statistical analyses. Positively stained cells per mm^2^/area were counted. 

### 2.4. Statistical Analyses

Proliferation, apoptosis, and DNA damage are expressed as a fold change (FC) relative to the control of each experiment ± standard error. To examine differences between micronutrient-treated and non-treated groups, generalised estimating equations (GEE) were used with log2-transformed positive stain per area to estimate fold change compared to controls. Independence correlation structure was assumed for the GEE to account for measurements from multiple regions per explant. Pre-specified post-hoc contrasts comparing individual sodium selenite and potassium iodide treatments with combination of sodium selenite and potassium iodide were performed with Bonferroni adjustment for multiple comparisons. P-values of less than 0.05 were considered significant. All analyses were performed using R version 3.5.3 or later.

## 3. Results

### 3.1. Confirmation of Micronutrient Uptake within the Treated First Trimester Placenta Explants

**Figure 1:**
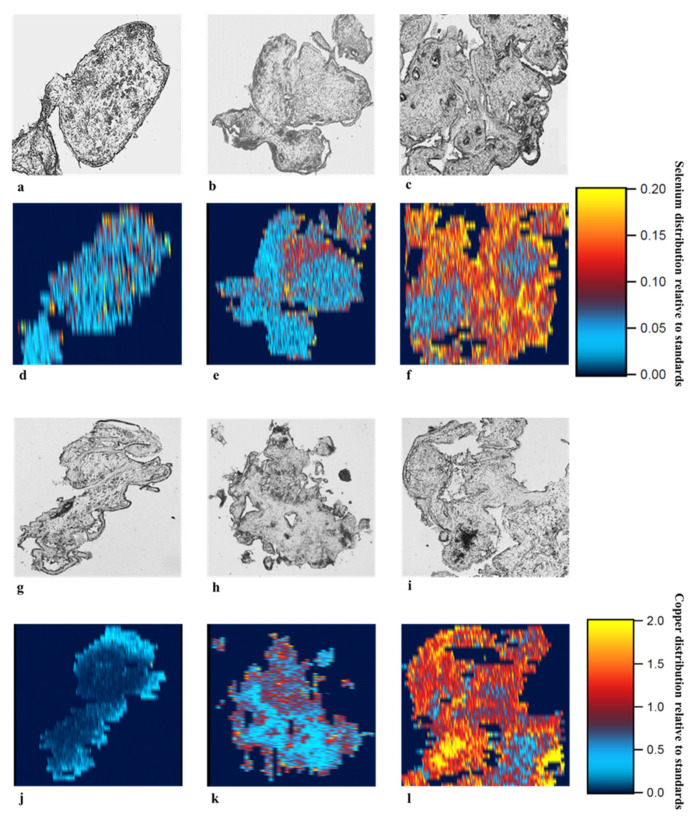
Selenium and copper uptake in first trimester human placental explants. Placenta explants (7–12 weeks of gestation) were supplemented with sodium selenite (**a** and **d**: sterile MilliQ water, **b** and **e**: 0.8 µM or **c** and **f**: 1.6 µM) or copper (II) sulfate (**g** and **j**: sterile MilliQ water, **h** and **k**: 20 µM or **i** and **l**: 40 µM) for 72 h (*n* = 3). Tissue sections (10 µm) were placed on microscope slides. Standards were made by dissolving 10% gelatine in 0, 1, 10, and 100 mM solutions of selenium and copper. Laser ablation inductively coupled plasma-mass spectrometry (LA-ICP-MS) analyses on the standard gelatine and placental explant sections were analysed using a Resolution 193 nm excimer laser ablation system coupled to an Agilent 7900x ICP-MS. Samples were ablated with a series of parallel lines: 23 μm spots size, 23 μm/s speed, 10 Hz repetition rate and a fluence of ~1 J/cm^2^. Intensity of micronutrients were recorded as counts per second for each isotope. Data was processed using the iolite data processing software, and element intensity calculated relative to the gelatine standards to form a 2D map of micronutrient distribution over a chosen surface of each placental explant sample.

Qualitative assessment of micronutrient distributions with laser ablation inductively coupled plasma-mass spectrometry (LA-ICP-MS) element imaging showed that supplementation with selenium and copper increased the content of these micronutrients in the placenta explants confirming the micronutrient uptake by explants from the media supplemented with selenium and copper (Figure 1; Supplementary Figures S1 and S2). In all three placentas, highest uptake of selenium was found in tissues treated with supraphysiological concentration of sodium selenite (1.6 µM) indicated by more red and yellow pixels in the LA-ICP-MS images (Figure 1d–f, Supplementary Figure S1d–f,j–l). In two placentas, it was apparent that with increasing selenium concentration, there was an increased uptake of selenium on the 2D map (Figure 1d–f and Supplementary Figure S1j–l). Assessment of tissues treated with copper (II) sulfate showed that control tissues had the lowest level of copper visualised by dark and light blue pixels. All tissues supplemented with physiological concentration (20 µM) copper (II) sulfate and two tissues supplemented with supraphysiological concentration of copper (II) sulfate (40 µM) (Figure 1j–l; Supplementary Figure S2d–f,j–l) had a higher content of copper compared to the control. This method was not able to assess iodine uptake.

### 3.2. Apoptosis and DNA Damage Induction by Menadione and Antimycin

A dose response experiment (*n* = 3) was performed to find the optimal concentration of menadione and antimycin for inducing oxidative stress in first trimester human placenta tissue. The following concentrations of menadione (0, 60, 120, and 240 µM) and antimycin (0, 240, 480, and 960 µM) were tested. Immunohistochemical analysis for DNA damage using 8-hydroxy-2’-deoxyguanosine was used to select the optimal concentration that induced sufficient DNA damage compared to vehicle control in the absence of micronutrient supplementation. The concentrations selected were 120 µM for menadione and 480 µM for antimycin. 

**Figure 2:**
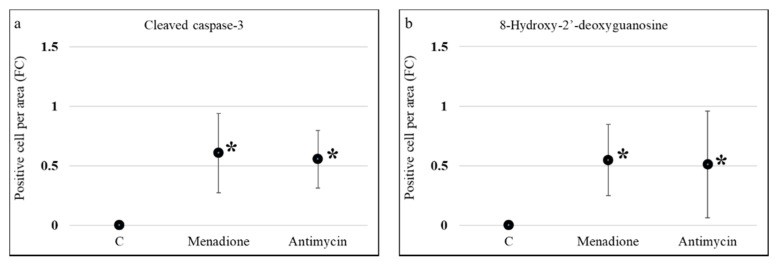
Effect of menadione and antimycin on apoptosis and DNA damage in first trimester human placental explants. Explants from 7–12 weeks of gestation (*n* = 12) were cultured for 5 days in media with 10% *v*/*v* FBS and 1% *v*/*v* Antibiotic-Antimycotic and then treated with 120 µM menadione, 480 µM antimycin or 0.1% ethanol (vehicle control) for 24 h. Immunohistochemical labelling for (**a**) apoptosis (cleaved caspase-3) or (**b**) DNA damage (8-hydroxy-2’-deoxyguanosine) was performed. Eight randomly selected regions per explant were used for quantification and statistical analyses. Data are presented as a fold change (FC) relative to control ± standard error. Statistical significance was assessed using Generalised Estimating Equations with independence correlation structure with log 2-transformed positive stain per area to estimate fold change compared to controls followed by pre-specified post-hoc Bonferroni adjusted multiple comparisons. * Indicates statistically different (*p* < 0.05) from control.

In the 12 first trimester placenta explants used in this study, immunohistochemistry analyses showed that compared to vehicle control (0.1 % ethanol) 120 µM menadione and 480 µM antimycin, in the absence of micronutrient supplementation, significantly increased the number of apoptotic cells and DNA damage (*p* < 0.05) (Figure 2 and Supplementary Figure S3). This confirmed that oxidative stress was induced in the cultured first trimester placenta explants and that this system could be used to assess whether micronutrient supplementation protects against induced oxidative stress.

### 3.3. Effect of Selenium, Iodine and Copper Supplementation on Proliferation

**Figure 3:**
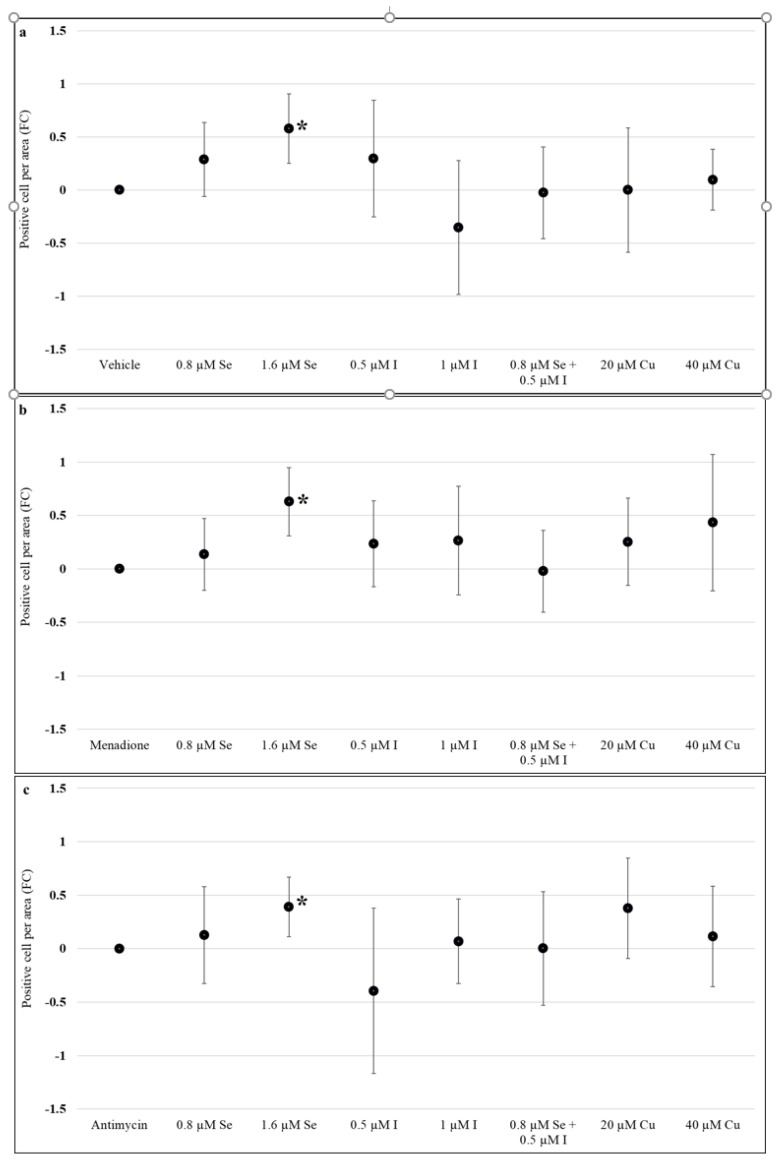
Effect of selenium, iodine, and copper supplementation on proliferation in first trimester placental explants. Placenta explants from 7 to 12 weeks of gestation (*n* = 12) were cultured for 48 h for syncytial regeneration followed by supplementation with sodium selenite (0, 0.8, or 1.6 µM), potassium iodide (0, 0.5, or 1 µM), combination of sodium selenite and potassium iodide (0.8 µM sodium selenite and 0.5 µM potassium iodide), or copper (II) sulfate (0, 20, or 40 µM) for 72 h with supplementation replenished every 24 h. (**a**) Placental explants were harvested following 72 h of supplementation and proliferation assessed by immunolabelling for Ki67. In addition, after 72 h supplementation, placental explants were treated with (**b**) 120 µM menadione or (**c**) 480 µM antimycin for 24 h to induce oxidative stress and then assessed for proliferation. Eight randomly selected regions per explant were used for quantification and statistical analyses. Data presented as a fold change (FC) relative to control ± standard error. Statistical significance was assessed using Generalised Estimating Equations with independence correlation structure with log 2-transformed positive stain per area to estimate fold change compared to controls followed by pre-specified post-hoc Bonferroni adjusted comparisons. * Indicates statistically different (*p* < 0.05) from vehicle control (C).

Ki67 positivity was used to assess whether any of the micronutrients altered proliferation within the treated placenta explant tissues in the absence of oxidative stress. There were no changes in proliferation except for treatment with 1.6 µM sodium selenite which increased cell proliferation after 72 h of supplementation (0.58, 95% CI: 0.25, 0.90) (Figure 3a). When oxidative stress was induced, with menadione or antimycin, there were no changes in proliferation except for supraphysiological concentration of selenium which again showed an increase in the number of Ki67 positive cells per mm^2^ after treatment with menadione (0.63, 95% CI: 0.31, 0.95) (Figure 3b), or antimycin (0.39, 95% CI: 0.11, 0.67) (Figure 3c) (*p* < 0.05).

### 3.4. Effect of Selenium, Iodine, and Copper Supplementation on Apoptosis

**Figure 4:**
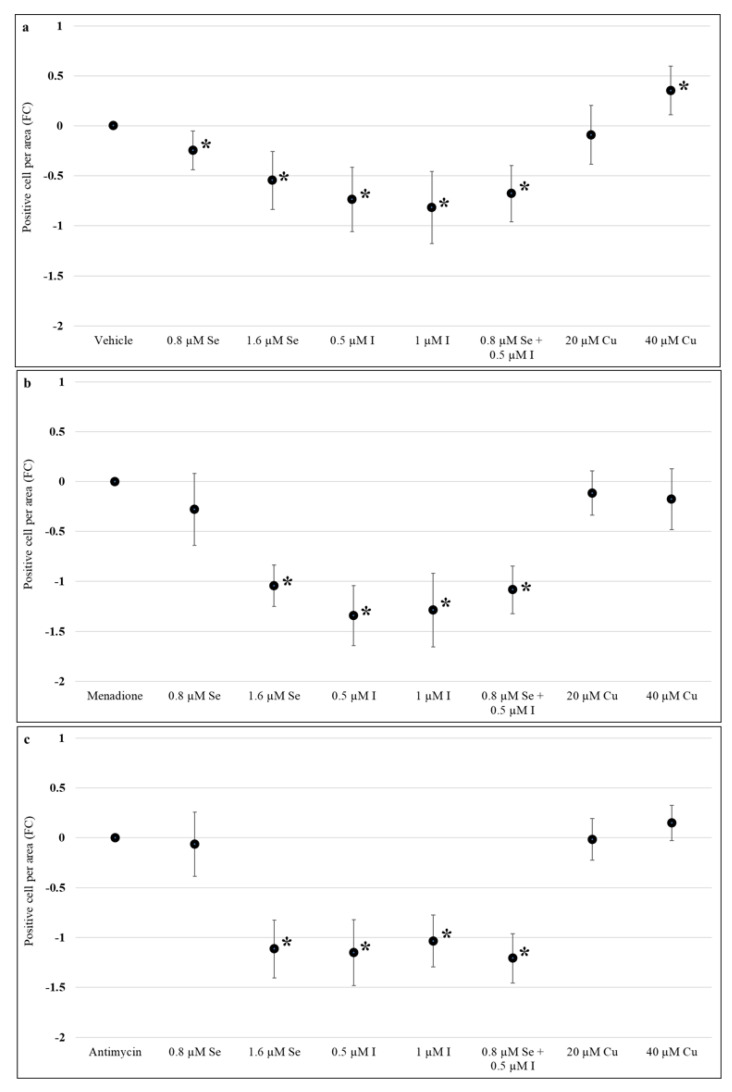
Effect of selenium, iodine, and copper supplementation on apoptosis in first trimester placental explants. Placenta explants from 7 to 12 weeks of gestation (*n* = 12) were cultured for 48 h for syncytial regeneration followed by supplementation with sodium selenite (0, 0.8, or 1.6 µM), potassium iodide (0, 0.5, or 1 µM), combination of sodium selenite and potassium iodide (0.8 µM sodium selenite and 0.5 µM potassium iodide), or copper (II) sulfate (0, 20, or 40 µM) for 72 h with supplementation replenished every 24 h. (**a**) Placental explants were harvested at the end of 72 h supplementation and apoptosis assessed by immunolabelling for cleaved caspase-3. In addition, after 72 h supplementation, placental explants were treated with (**b**) 120 µM menadione or (**c**) 480 µM antimycin for 24 h to induce oxidative stress and then assessed for apoptosis. Eight randomly selected regions per explant were used for quantification and statistical analyses. Data presented as a fold change (FC) relative to control ± standard error. Statistical significance was assessed using Generalised Estimating Equations with independence correlation structure with log 2-transformed positive stain per area to estimate fold change compared to controls followed by pre-specified post-hoc Bonferroni adjusted comparisons. * Indicates statistically different (*p* < 0.05) from control.

In the absence of oxidative stress, both 0.8 and 1.6 µM sodium selenite reduced apoptosis (−0.25, 95% CI: −0.44, −0.053 and −0.54, 95% CI: −0.83, −0.25, respectively) compared to control (sterile MilliQ water) (Figure 4a). Potassium iodide (0.5, 1 µM) significantly reduced apoptosis (−0.73, 95% CI: −1.06, −0.41 and −0.82, 95% CI: −1.18, −0.46, respectively) (Figure 4a). The combination of 0.8 µM sodium selenite and 0.5 µM potassium iodide also reduced apoptosis compared to the vehicle control (−0.68, 95% CI: −0.96, −0.4) (*p* < 0.05) (Figure 4a). In addition, the effect of the combination of sodium selenite and potassium iodide in apoptosis reduction was significantly more than individual supplementation with 0.8 µM sodium selenite (*p* < 0.05) but not 0.5 µM potassium iodide. Treatment with supraphysiological concentration of copper (II) sulfate (40 µM) significantly increased apoptosis (0.35, 95% CI: 0.11, 0.60) compared to control (Figure 4a). 

Assessment of apoptosis following induction of oxidative stress by menadione showed that 1.6 µM sodium selenite (−1.04, 95%CI: −1.25, −0.84), both 0.5 µM (−1.34, 95%CI: −1.64, −1.04) and 1 µM potassium iodide (−1.28, 95%CI: −1.65, −0.91), and the combination of 0.8 µM sodium selenite and 0.5 µM potassium iodide (−1.1, 95%CI: −1.32, −0.84) were associated with a significant reduction in apoptosis (*p* < 0.05) when compared to the menadione alone control (Figure 4b). Combination of sodium selenite and potassium iodide reduced apoptosis more than 0.8 µM sodium selenite (*p* < 0.05) but not more than potassium iodide (0.5 µM) alone (Figure 4b). Copper (II) sulfate did not significantly impact apoptosis (*p* > 0.05) (Figure 4b). Similar results were seen when oxidative stress was induced using antimycin (Figure 4c), that is, reduction in apoptosis with the higher dose of sodium selenite (1.6 µM), both potassium iodide concentrations (0.5 and 1 µM) and sodium selenite and potassium iodide combination but no changes in apoptosis with copper (II) sulfate compared to the antimycin alone control (Figure 4c).

### 3.5. Effect of Selenium, Iodine and Copper Supplementation on DNA Damage

**Figure 5:**
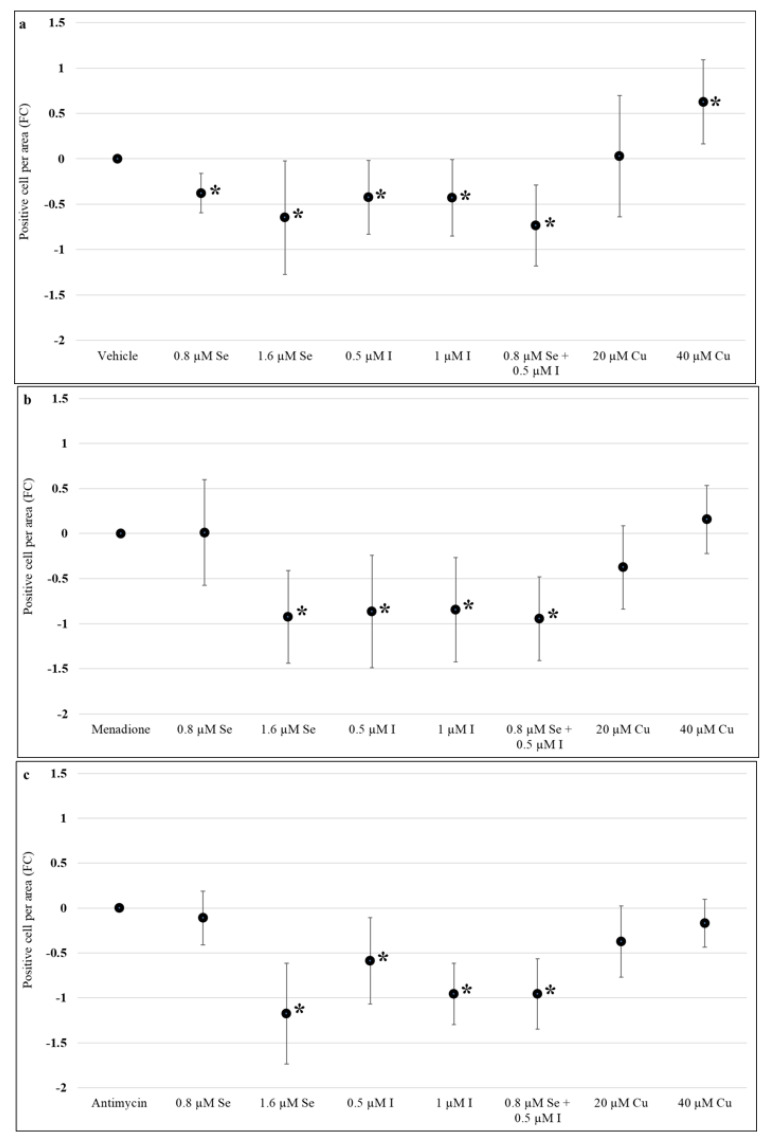
Effect of selenium, iodine, and copper supplementation on DNA damage in first trimester human placental explants. Placenta explants from 7 to 12 weeks of gestation (*n* = 12) were cultured for 48 h for syncytial regeneration followed by supplementation with sodium selenite (0.8 or 1.6 µM), potassium iodide (0.5 or 1 µM), combination of sodium selenite and potassium iodide (0.8 µM sodium selenite and 0.5 µM potassium iodide), or copper (II) sulfate (20 or 40 µM) for 72 h with supplementation replenished every 24 h. (**a**) Placental explants were harvested following 72 h of supplementation and DNA damage assessed by immunolabelling for 8-hydroxy-2′-deoxyguanosine. After 72 h of supplementation, placental explants were treated with (**b**) 120 µM menadione or (**c**) 480 µM antimycin for 24 h to induce oxidative stress and then assessed for DNA damage. Eight randomly selected regions per explant were used for quantification and statistical analyses. Data presented as a fold change (FC) relative to control ± standard error. Statistical significance was assessed using Generalised Estimating Equations (GEE) with independence correlation structure with log 2-transformed positive stain per area to estimate fold change compared to controls followed by pre-specified post-hoc Bonferroni adjusted comparisons. * Indicates statistically different (*p* < 0.05) from control.

Both concentrations of sodium selenite 0.8 µM (−0.4, 95% CI: −0.6, −0.16) and 1.6 µM (−0.65, 95% CI: −1.27, −0.02), potassium iodide 0.5 µM (−0.42, 95% CI: −0.83, −0.02), 1 µM (−0.43, 95% CI: −0.85, 0.01), and the combination of 0.8 µM sodium selenite and 0.5 µM potassium iodide (−0.73, 95% CI: −1.18, −0.29) reduced DNA damage compared to control (sterile MilliQ water) following 72 h of treatment (*p* < 0.05) (Figure 5a). However, the effect of the combination of sodium selenite and potassium iodide was not significantly different from individual supplementation with sodium selenite or potassium iodide (*p* > 0.05) (Figure 5a). There was a significant increase in DNA damage in cells treated with super physiological concentration of copper (II) sulfate (0.63, 95% CI: 0.17, 1.09) (Figure 5a). 

Assessment of DNA damage after oxidative stress induction with menadione showed that 1.6 µM sodium selenite (−0.92, 95% CI: −1.44, −0.41), both concentrations of potassium iodide: 0.5 µM (−0.87, 95% CI: −1.49, −0.24), 1 µM (−0.85, 95% CI: −1.42, −0.27) and the combination of sodium selenite and potassium iodide (−0.95, 95% CI: −1.41, −0.48) resulted in a significant reduction in DNA damage compared to the menadione alone control while copper (II) sulfate did not have any significant effect (*p* > 0.05) (Figure 5b). While the combination of 0.8 µM sodium selenite and 0.5 µM potassium iodide significantly reduced DNA damage after menadione treatment, there was no effect with the individual supplementation of 0.8 µM sodium selenite (*p* > 0.05). In addition, no significant difference was found between supplementation with 0.5 µM potassium iodide and the combination of 0.8 µM sodium selenite and 0.5 µM potassium iodide (*p* > 0.05) (Figure 5b). Similar results were seen in DNA damage assessment of placenta explants treated with antimycin (Figure 5c).

## 4. Discussion

Selenium supplementation increased proliferation and reduced DNA damage and apoptosis in the absence or presence of oxidative stress. This can be explained by the effect of selenium on the cell cycle and the antioxidant system [31,32]. Selenium stimulates the transition from Gap 2 to the mitosis phase in the cell cycle and this increases cell division or proliferation [32]. The cell cycle is precisely monitored and damaged DNA molecules can be detected at several checkpoints [33]. Although checkpoints in different cell cycle phases may work differently, the ultimate outcome is if the DNA molecule is not repaired the cell containing damaged DNA cannot proliferate and undergoes apoptosis [33]. GPx 4 and TRx are seleno-antioxidant enzymes that can protect cells against oxidative damage such as DNA damage, therefore reducing apoptosis [34,35,36]. Supraphysiological levels of selenium increased proliferation and decreased apoptosis in first trimester placental tissues [28]. Notably, when placenta explants were treated with menadione or antimycin the lower concentration of sodium selenite could not reduce DNA damage or apoptosis. This suggests that under conditions of oxidative stress, cells may require a higher concentration of selenium, that is, supraphysiological levels of selenium, for protection against oxidative damage.

This study showed that potassium iodide could protect from oxidative damage to DNA molecules and reduced apoptosis. This is consistent with previous findings showing the protective effect of potassium iodide against oxidative stress in a placenta cell line [28,37]. Systemically, iodine is a radical scavenger as seen by its ability to metabolise H_2_O_2_ in thyroid hormone production [38]. The exact mechanisms by which iodine interacts with the antioxidant system in combating oxidative stress has not been clearly defined but studies have shown that iodine has antioxidant properties such as increasing total antioxidant status of human serum [39]. Iodine sufficient pregnant women (>150 µg/L urinary iodine concentration) had higher activity of the enzyme SOD compared to iodine deficient women [24]. Interestingly, the combination of sodium selenite and potassium iodide resulted in a greater reduction in DNA damage in antimycin treated cells, compared to either micronutrient alone. This suggests that combining iodine and selenium may provide a stronger protection to the placenta against oxidative stress. Iodide and selenium can form selenenyl iodide that is a substrate for placental TRx and may increase antioxidant activity of TRx [40], thereby reducing DNA damage and apoptosis. 

In a prospective cohort study of 1065 pregnant Australian women, a high level of maternal plasma copper was associated with higher plasma C-reactive protein (CRP) concentrations [3]. CRP is an inflammatory biomarker that increases in oxidative stress [41] and its higher levels are associated with a greater incidence of pregnancy complications such as preterm birth [42] and preeclampsia [43]. The results of this study showed that a high concentration of copper increased DNA damage and apoptosis. Excess intake of copper may increase production of reactive oxygen species and diminish antioxidant defence systems [44], resulting in DNA damage and apoptosis. Interestingly, in menadione or antimycin induced oxidative stress the toxic effect of excess copper was not apparent. To remove reactive molecules and protect the cells against oxidative stress, the antioxidant system is overexpressed and the activity of enzymes such as SOD1 increases [45]. Therefore, these enzymes may require higher levels of cofactors including copper. This avoids copper being accumulated and causing toxic effects. 

We could only examine the effect of three essential micronutrients on oxidative stress in first trimester human placenta. Continued research on a group of micronutrients in placenta samples from other trimesters can address the potential differences across gestation. Using laser ablation inductively coupled plasma-mass spectrometry in the assessment of micronutrients uptake is novel and to the best of our knowledge, this is the first study that has directly mapped selenium and copper content and distribution in cultured placental explants. However, this method cannot confirm iodine uptake. Further research, including coupling this method with immunolabeling of different placental cells as well as quantifying micronutrients content within the placental explants, may provide a better understanding of the effect of micronutrients on the placenta.

## 5. Conclusions

In conclusion, selenium and iodine may protect the first trimester human placenta against oxidative stress in vitro. An excess intake of copper is related to oxidative damage to DNA and apoptosis. An optimal level of micronutrients may help to ensure healthy placental development.

## Figures and Tables

**Table 1:** Antibodies used for immunohistochemical labelling.

Antibody	Dilution	Target Species	CAT #	Company	Diluent	Antigen Retrieval
**Ki67**	1/100	Rabbit	ab16667	Abcam^®^	5% Goat serum	Citrate buffer (10 mM Citric acid; pH 6.0; 10 min boiling in microwave, Sixth Sense, Whirlpool, VIC, Australia)
**cleaved caspase-3**	1/100	Rabbit	CST.9661L	Cell Signalling Technology^®^	5% Goat serum	Citrate buffer (10 mM Citric acid; pH 6.0; 10 min boiling in microwave)
**8-hydroxy-2’-deoxyguanosine**	1/200	Mouse	ab48508	Abcam^®^	5% Goat serum	Citrate buffer (10 mM Citric acid; pH 6.0; 10 min boiling in microwave)

# refers to number.

## Data Availability

Data and material are available for data transparency.

## Supplementary Materials

The following are available online at https://www.mdpi.com/2072-6643/13/3/800/s1, Figure S1: Selenium uptake in first trimester human placental explants, Figure S2: Copper uptake in first trimester human placental explants, Figure S3: Apoptosis and DNA damage induction by menadione in first trimester human placental explants.
