# Supplementary material for: Effect of Selenium and Iodine on Oxidative Stress in the First Trimester Human Placenta Explants
Source: Nutrients. 2021 Feb 28;13(3):800. doi: 10.3390/nu13030800 (PMC7997475; doi:10.3390/nu13030800)
Supplement: Supplementary file 1 [file nutrients-13-00800-s001.pdf]

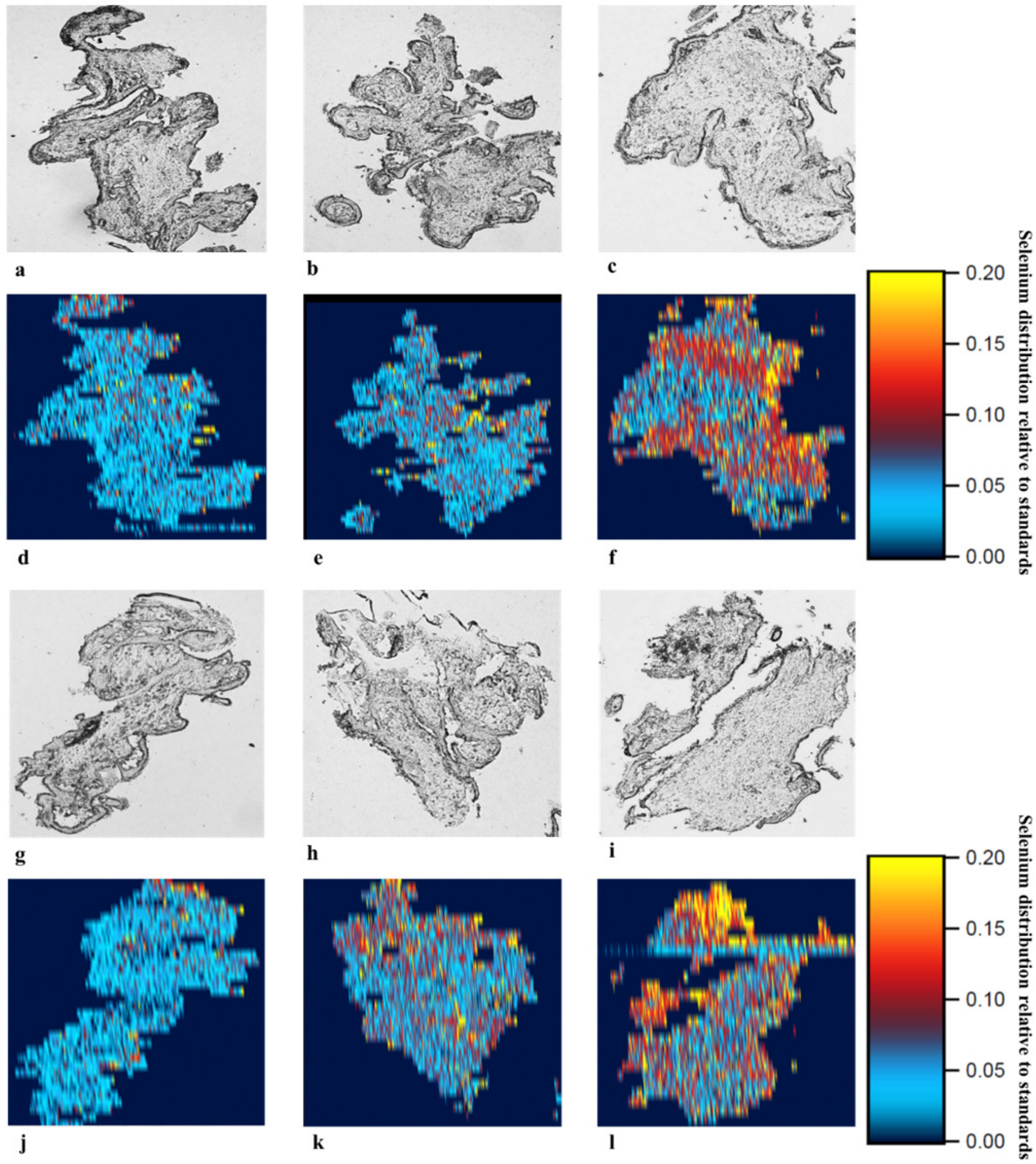

Supplementary Figure 1. Selenium uptake in first trimester human placental explants

Placenta explants from 7-12 weeks of gestation (N=12) were supplemented with sodium selenite (a & d & g & j: sterile MilliQ water, b & e & h & k: 0.8 µM or c & f & i & l: 1.6 µM) for 72 hours. Tissue sections were cut at 10 µm and placed on microscope slides. Standards were made by dissolving 10% gelatine in solutions of selenium and copper with 0, 1, 10, and 100 mM concentrations. Laser ablation inductively coupled plasma-mass spectrometry (LA-ICP-MS) analyses on the standard gelatine and placental explant sections were analysed at Adelaide Microscopy, University of Adelaide, using a Resolution 193 nm excimer laser ablation system coupled to an Agilent 7900x ICP-MS. Samples were ablated with a series of parallel lines: 23 µm spots size, 23 µm/s speed, 10 Hz repetition rate and a fluence of ~1 J/cm^2. Intensity of micronutrients were recorded as counts per second for each isotope. Data was processed using the iolite data processing software, and element intensity calculated relative to the gelatine standards to form a 2D map of micronutrient distribution over a chosen surface of each placental explant sample.

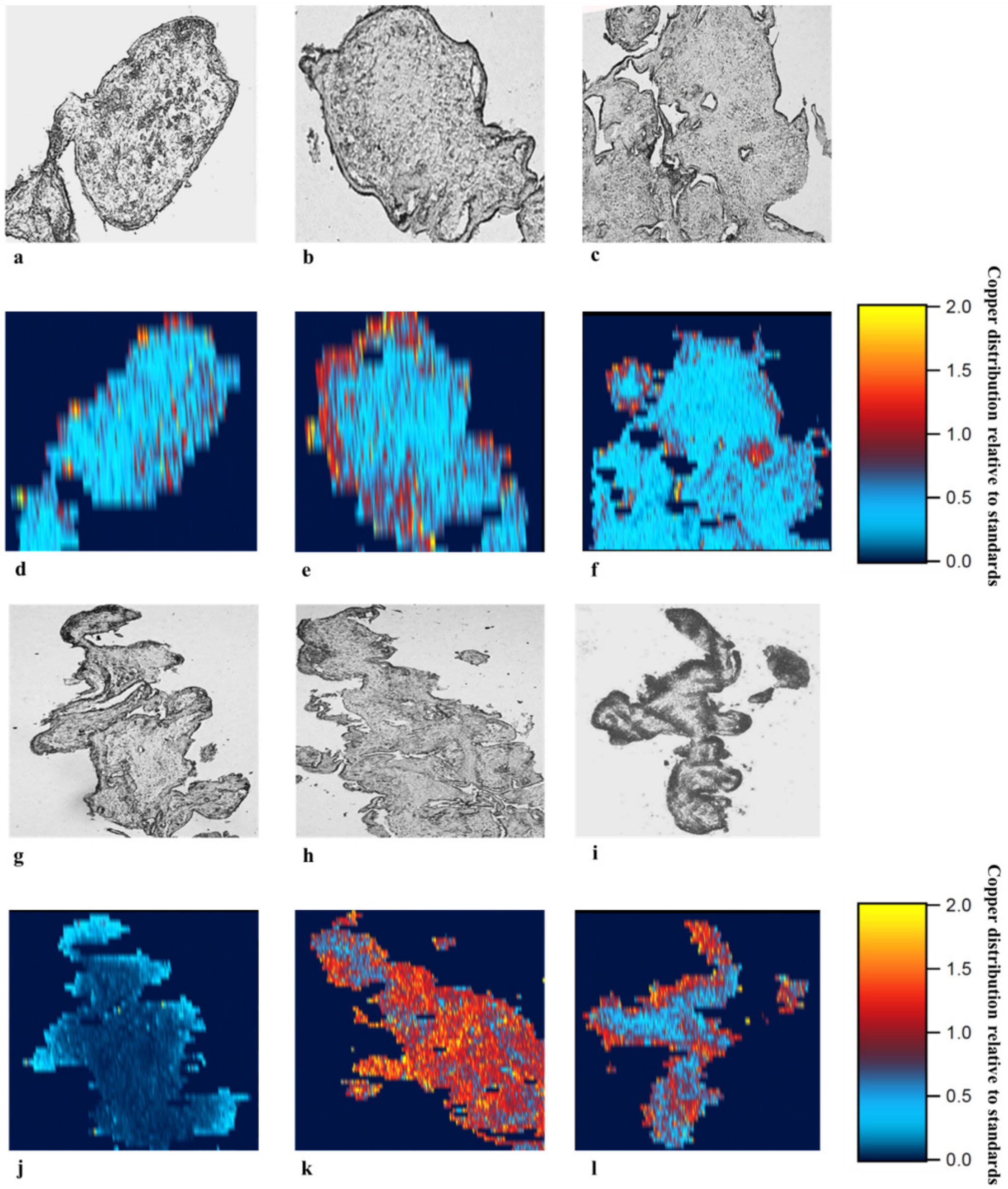

Supplementary Figure 2. Copper uptake in first trimester human placental explants

Placenta explants from 7-12 weeks of gestation (N=12) were supplemented with copper (II) sulfate (a & d & g & j: sterile MilliQ water, b & e & h & k: 20 µM or c & f & i & l: 40 µM) for 72 hours. Tissue sections were cut at 10 µm and placed on microscope slides. Standards were made by dissolving 10% gelatine in solutions of selenium and copper with 0, 1, 10, and 100 mM concentrations. Laser ablation inductively coupled plasma-mass spectrometry (LA-ICP-MS) analyses on the standard gelatine and placental explant sections were analysed at Adelaide Microscopy, University of Adelaide, using a Resolution 193 nm excimer laser ablation system coupled to an Agilent 7900x ICP-MS. Samples were ablated with a series of parallel lines: 23 µm spots size, 23 µm/s speed, 10 Hz repetition rate and a fluence of ~1 J/cm^2. Intensity of micronutrients were recorded as counts per second for each isotope. Data was processed using the iolite data processing software, and element intensity calculated relative to the gelatine standards to form a 2D map of micronutrient distribution over a chosen surface of each placental explant sample.

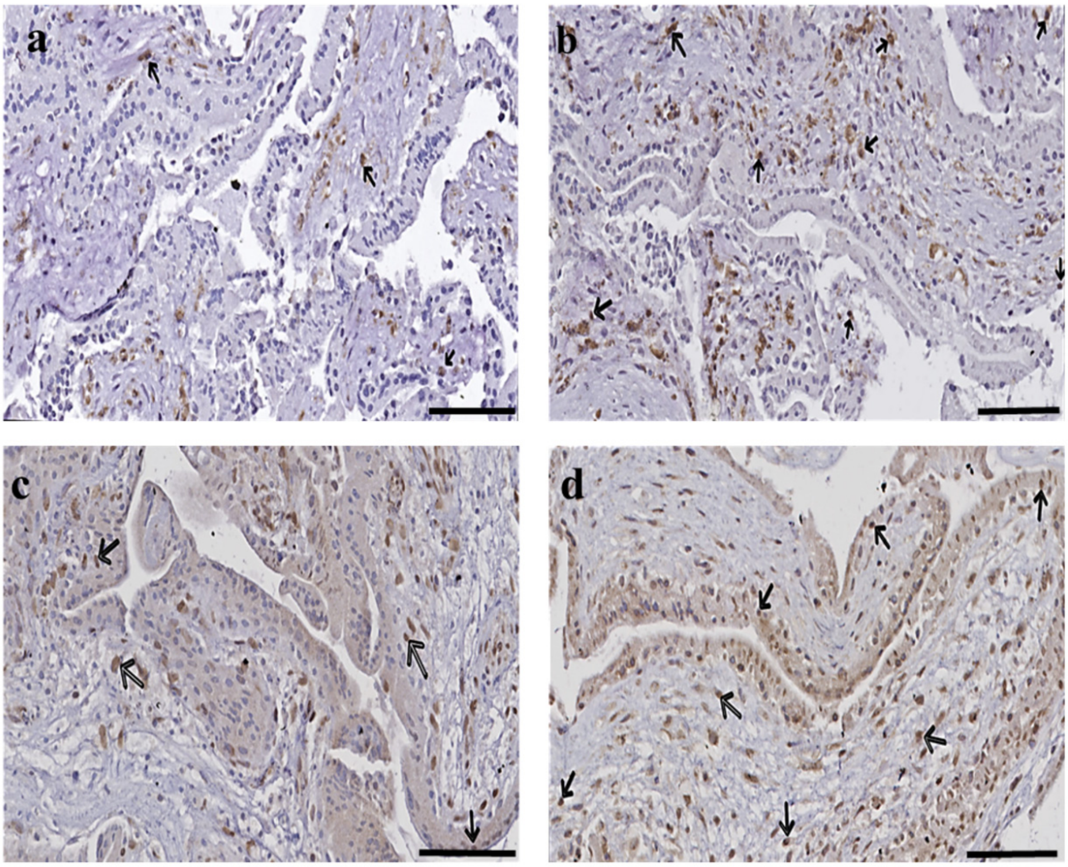

Supplementary Figure 3. Apoptosis and DNA damage induction by menadione in first trimester human placental explants

First trimester placenta tissues were cultured for 5 days in media including 10% v/v FBS and 1% v/v Antibiotic-Antimycotic and then treated with a) and c) 0.1% ethanol (vehicle control) or b) and d) 120 μM menadione for 24 hours (N=12). Immunohistochemical labelling for a) and b) apoptosis (cleaved caspase-3) or c) and d) DNA damage (8-hydroxy-2'-deoxyguanosine) was performed. Stained slides were scanned with a Hamamatsu NanoZoomer Digital Pathology slide scanner and NDP.view2 software were used for analyses. Black arrows indicate positively stained cells. Scale bar is 100 μm.
